# Synthesis, Study, and Discrete Dipole Approximation Simulation of Ag-Au Bimetallic Nanostructures

**DOI:** 10.1186/s11671-016-1435-4

**Published:** 2016-04-19

**Authors:** Yang Hu, An-Qi Zhang, Hui-Jun Li, Dong-Jin Qian, Meng Chen

**Affiliations:** Department of Chemistry, Shanghai Key Laboratory of Molecular Catalysis and Innovative Materials, Fudan University, Shanghai, 200433 P. R. China; Department of Materials Science, Fudan University, Shanghai, 200433 P. R. China

**Keywords:** Alloy, Bimetallic nanostructures, Core-shell, Discrete dipole approximation

## Abstract

Water-soluble Ag-Au bimetallic nanostructures were prepared via co-reduction and seed-mediated growth routes employing poly-(4-styrenesulfonic acid-co-maleic acid) (PSSMA) as both a reductant and a stabilizer. Ag-Au alloy nanoparticles were obtained by the co-reduction of AgNO_3_ and HAuCl_4_, while Ag-Au core-shell nanostructures were prepared through seed-mediated growth using PSSMA-Au nanoparticle seeds in a heated AgNO_3_ solution. The optical properties of the Ag-Au alloy and core-shell nanostructures were studied, and the growth mechanism of the bimetallic nanoparticles was investigated. Plasmon resonance bands in the range 422 to 517 nm were observed for Ag-Au alloy nanoparticles, while two plasmon resonances were found in the Ag-Au core-shell nanostructures. Furthermore, discrete dipole approximation theoretical simulation was used to assess the optical property differences between the Ag-Au alloy and core-shell nanostructures. Composition and morphology studies confirmed that the synthesized materials were Ag-Au bimetallic nanostructures.

**Electronic supplementary material:**

The online version of this article (doi:10.1186/s11671-016-1435-4) contains supplementary material, which is available to authorized users.

## Background

Noble metal nanoparticles (NPs) have recently attracted great attention due to their special electronic, optical, magnetic, and catalytic properties, which vary considerably from that of their bulk materials. Moreover, bimetallic NPs such as Ag-Au alloys and core-shell structures exhibit characteristic properties attributed to the synergic effect between the two metals and not observed in monometallic Au and Ag NPs [1]. The Au:Ag molar ratios and their geometrical arrangement have a significant influence on the resulting optical properties of bimetallic nanostructures [2]. Studies have shown that the surface plasmon resonance of bimetallic Ag-Au NPs could be adjusted within the range 400–520 nm, while for spherical Ag or Au NPs, these can be restricted to approximately 400 or 520 nm, respectively [3]. In addition, bimetallic NPs have excellent properties for surface-enhanced Raman spectroscopy, which can be exploited in potential bioanalytical and biomedical applications [4]. Ag-Au alloy NPs are also more catalytically active than their counterparts in catalytic reactions such as CO oxidation [5]. Thus, the synthesis of bimetallic Ag-Au NPs with controlled structures and properties is relevant for various applications.

Over the past decade, there has been substantial interest in the preparation of Ag-Au core-shell and alloy nanostructures through methods such as the simultaneous co-reduction of Au and Ag salts in solution or seed-mediated growth through the deposition of metal nanostructures on the surface of metallic seeds. Chemical-reducing agents such as sodium borohydride [6–8], hydroxylamine hydrochloride [4, 9, 10] ascorbic acid [1, 11], formaldehyde [12], and citrate [13] have been commonly used in the co-reduction of Au and Ag. Wilson et al. [2] used sodium borohydride as a reductant to synthesize dendrimer-encapsulated bimetallic Ag-Au alloy and core-shell NPs (1–3 nm in size). Cheng et al. [14] utilized ascorbic acid in the synthesis of star-shaped Ag-Au bimetallic NPs. Additionally, radiation methods, including γ-ray, ultraviolet light, and microwave, have also been frequently considered. Hodak et al. [15] reported on the laser-assisted synthesis of Ag-Au core-shell structures through seed-mediated growth, whereas Gonzalez et al. [16] utilized an ultraviolet initiator to produce Ag-Au alloy and core-shell bimetallic NPs.

In the present study, Ag-Au alloy and core-shell nanostructures with a plasmon resonance absorption within the range 400–520 nm were successfully synthesized through co-reduction and seed-mediated growth methods. The co-reduction method was mainly employed to prepare Ag-Au alloy nanostructures by direct mixing of the metal salts. In the seed-mediated growth method, the cores were initially prepared, and the shells were then deposited on the surface of the core seeds using poly-(4-styrenesulfonic acid-co-maleic acid) (PSSMA) as the reductant and stabilizer. Comparison of the two synthetic methods allowed assessment of the growth mechanism of Ag-Au alloy and core-shell nanostructures. In addition, the plasmon resonance absorptions were examined through theoretical extinction spectra simulated by the discrete dipole approximation (DDA) model. The surface plasmon resonances of Ag-Au core-shell and alloy NPs were found to be similar to those of monometallic Ag or Au NPs [17].

## Methods

### Materials and Synthesis

PSSMA sodium salt, with a styrenesulfonic acid to maleic acid ratio of 3:1 and average molecular weight of 20,000 gmol^−1^, was purchased from Sigma-Aldrich. AgNO_3_, HAuCl_4_, and NH_3_H_2_O (25 %) were obtained from the Shanghai Chemicals Co. All reagents were used as received without further purification. The solvent was deionized with water purified by a Millipore system.

Ag-Au alloy and core-shell NPs were prepared by the seed-mediated growth method. In detail, PSSMA-stabilized Ag and Au NPs (PSSMA-Ag NPs and PSSMA-Au NPs, respectively) were initially prepared through a hydrothermal method detailed below. HAuCl_4_ and AgNO_3_ salts were then separately added into the PSSMA-Ag NP or PSSMA-Au NP seed solutions, respectively, to obtain Ag-Au alloy and core-shell NPs.

#### Synthesis of PSSMA-Stabilized Ag-Au Alloy NPs

In a typical synthesis of PSSMA-stabilized Ag-Au alloy NPs, 20 mL of aqueous solution of PSSMA (40 mM, calculated in terms of the repeating unit) was added to 20 mL of AgNO_3_ solution (2.5 mM). The pH value of the resulting solution was adjusted to 10 by addition of NH_3_H_2_O. The final concentrations of AgNO_3_ and the PSSMA repeating unit were 1.25 and 20 mM, respectively. The mixture was then loaded into an autoclave and heated at 120 °C for 12 h.

After the reaction, 4.4 mL of the synthesized PSSMA-Ag NPs was diluted to 40 mL in a three-neck flask to yield a 0.14-mM PSSMA-Ag NPs suspension. An appropriate amount of HAuCl_4_ salts (4 wt%) was injected into the suspension. The mixture was then heated in an oil bath at 90 °C for 19 h. The Ag^+^:AuCl_4_^−^ molar ratio was adjusted to 1:2, 1:1, 1:0.5, 1:0.333, 1:0.2, and 1:0.125. During the reaction, 2 mL aliquots of the NP suspension was retrieved at 10, 20, 40, 60, 90, 120 min, 4, and 19 h and cooled in an ice bath for UV–Vis absorption characterization.

#### Synthesis of PSSMA-Stabilized Ag-Au Core-Shell NPs

In a typical synthesis of PSSMA-stabilized Ag-Au core-shell NPs, 39.8 mL of deionized water was added into a beaker with 0.294 g of PSSMA. Following dissolution of the PSSMA, 0.2 mL of HAuCl_4_ (4 wt%) was injected into the beaker. The pH value of the resulting solution was approximately 6.5. The final concentrations of HAuCl_4_ and the PSSMA repeating unit were 0.5 and 9.45 mM, respectively. The mixture was heated in the three-neck flask at 90 °C for 3 h.

After the reaction, 20 mL of the synthesized PSSMA-Au NP suspension was added into another three-neck flask. A different amount of AgNO_3_ salts was then injected into the suspension. The mixture was heated in an oil bath at 90 °C for 25 h. The Ag^+^:AuCl_4_^−^ molar ratio was adjusted to 1:0.17, 1:0.5, 1:1, 1:2, and 1:4. In order to monitor the formation process of the bimetallic NPs, 2 mL aliquots of the NP dispersion was retrieved at 1, 2, 4, 6, 9, 12, and 25 h and cooled in an ice bath for UV–Vis absorption characterization.

### Characterization and Measurements

UV–Vis absorption spectra were recorded on a UV-2550 spectrophotometer (Shimadzu, Japan) at room temperature using a glass cuvette with a 1-cm optical path, the wavelength of which varied between 200 and 800 nm. X-ray photoelectron spectra (XPS) measurements were performed on a VGESCALAB MKII X-ray photoelectron spectrometer, using non-monochromatized Mg-Kα X-rays as the excitation source. The binding energies obtained in the XPS analysis were corrected by referencing the C_1s_ peak to 284.60 eV. X-ray diffraction was carried out using a Bruker D8 advance X-ray diffractometer with Cu-Kα radiation (*λ* = 1.54056 Å). Samples for measurement were prepared by placing bimetallic colloid solution droplets on quartz plates and allowing them to dry at 50 °C and repeating it for three times.

The selected area electron diffraction pattern, elemental analysis, and transmission electron microscopy (TEM) images were acquired on a JEOL JEM-2010 transmission electron microscope, operating an accelerating voltage of 200 kV and fitted with an energy-dispersive X-ray emission analyzer. All TEM samples were made using aqueous colloids of the metal NPs directly without size selection. The NPs were deposited onto Formvar-coated 230-mesh copper grids. Particle size distribution analysis was carried out by manually digitizing the TEM image with ImageJ software, from which the average size and standard deviation of metal NPs could be calculated.

## Results and Discussion

### Optical Properties and DDA Simulation of Ag-Au Alloy NPs

Highly stable Ag-Au alloy NPs with varied molar ratios were synthesized through the thermal reaction of PSSMA-Ag NPs and HAuCl_4_ solutions at 90 °C. The color of the Ag NP colloids changed from deep brown to pale yellow upon addition of HAuCl_4_ and subsequently to light red 10 min after. In addition, the reddish color gradually deepened as the reaction proceeded or with an increase in the Au:Ag molar ratio.

**Fig. 1:**
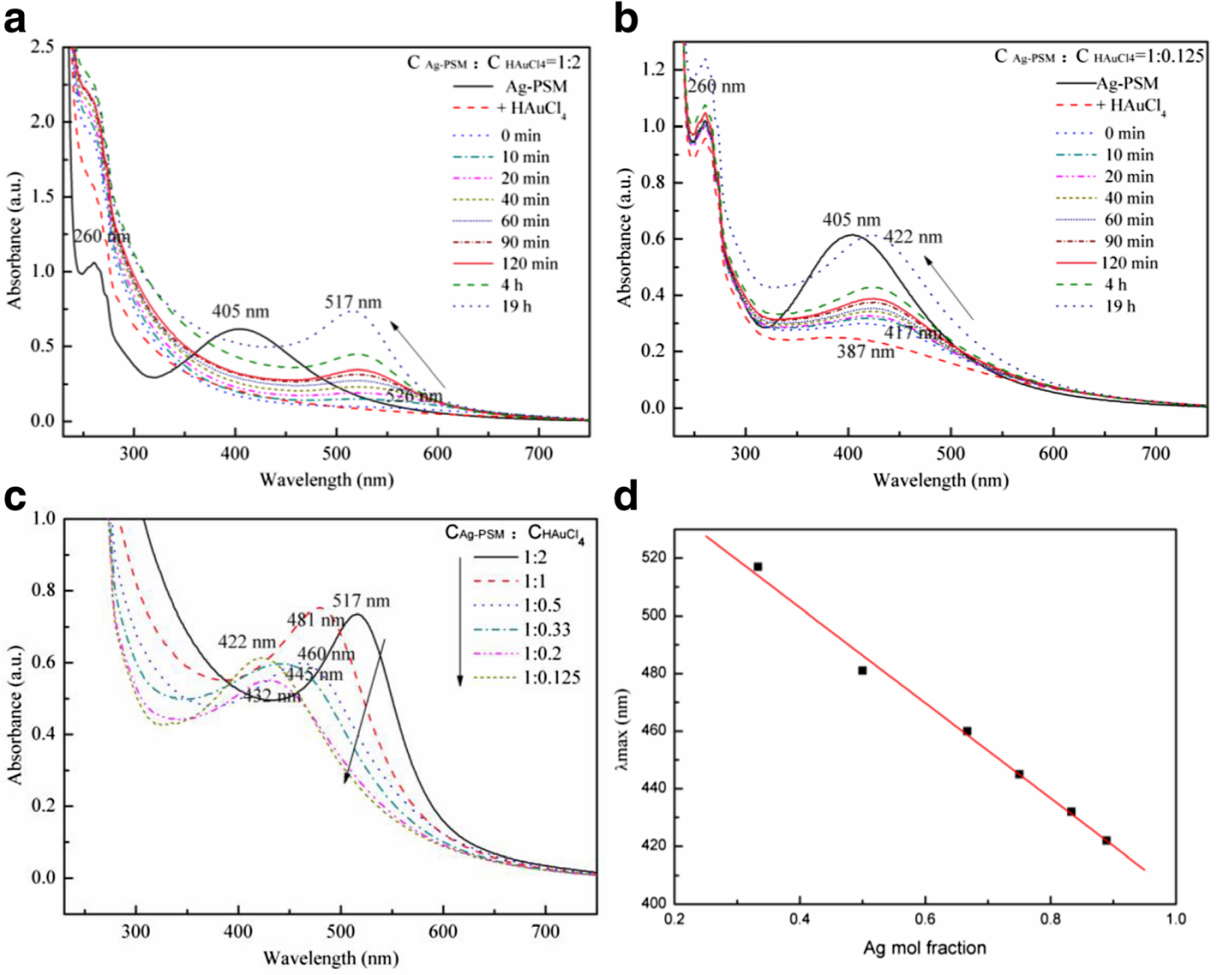
Time-dependent UV–Vis spectra of Ag-Au alloy nanoparticles (NPs) prepared from PSSMA-Ag NPs:HAuCl_4_ at molar ratios **a** 1:2 and **b** 1:0.125. **c** UV–Vis absorption spectra of Ag-Au alloy NPs at different Ag mole fractions. **d** Plot of the wavelength corresponding to the maximum absorbance against the Ag mole fraction for Ag-Au alloy NPs obtained by varying the mole fractions of HAuCl_4_ while keeping the concentration of Ag NPs constant (C_PSSMA-Ag NPs_ = 0.14 mM). The *solid line* is a linear fit of the absorption maximum to the increasing Ag mole fraction. The *squares* correspond to the experimental data

Figure 1 shows the time-dependent UV–Vis absorption spectra of the Ag-Au alloy nanostructures obtained with the Ag^+^:AuCl_4_^−^ molar ratios of 1:2, 1:1, 1:0.5, 1:0.33, 1:0.2, and 1:0.125. The single and symmetric peaks centered at 405 nm were attributed to the primary dipolar excitation of PSSMA-Ag NPs, which disappeared upon the addition of AuCl_4_^−^ solution. This change is attributed to the galvanic replacement reaction, namely, Ag(NP) + AuCl_4_^−^→Au(NP) + Ag^+^ [18, 19].

When the mixture was heated at 90 °C for 10 min, a new absorption peak began to evolve at a longer wavelength, nearer to the characteristic absorption peak of Au NPs. Continued heating lead to a slow increase in the absorption intensity and a successive blueshift of the surface plasmon resonance bands, indicating that the synthesized NPs were mostly composed of elemental Au. In addition, with an increasing Ag:Au molar ratio, the position of the final absorption band was closer to the absorption peak of Ag NPs (compare Fig. 1a and Fig.1b). After heating for 19 h, the final absorption bands were located at 517, 481, 460, 445, 432, and 422 nm, respectively (Fig. 1c). Further, a linear relationship between the resonance locations and the Ag mole fraction could be observed (the linear correlation coefficient was 0.99; Fig. 1d), indicating the successful formation of composites. The plasmon resonance absorptions of bimetallic nanocrystals vary considerably from those of their monometallic NP counterparts since their surface plasmon polaritons are determined by two different dielectric functions [20]. As reported in previous studies [13, 16], the formation of Ag-Au alloy structures can be confirmed by the presence of one absorption band, which would blueshift with an increase in the molar ratio of Ag [21–23] contrary to what would be observed in the formation of core-shell nanostructures.

**Fig. 2:**
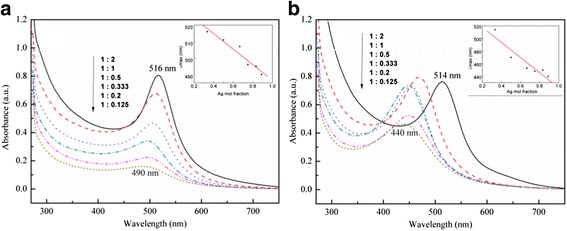
UV–Vis absorption spectra of Ag-Au alloy samples prepared at 90 °C for 19 h with different molar ratios of Ag^+^:AuCl_4_^−^ in the solutions **a** without adjusting the pH and **b** at pH 10. The *inset* shows the linear relationship corresponding to the maximum absorption wavelength. The Ag concentration in the PSSMA-Ag NPs corresponds to 0.14 mM

Alloy NPs were also prepared through co-reduction of Ag and Au salt solutions at various pHs in an autoclave heated at 90 °C. In our previous work [24], it was established that a pH value of 10 is suitable for the synthesis of Ag NPs. UV–Vis absorption spectra of the samples (Fig. 2) clearly indicate that the plasmon resonance bands changed when the pH was adjusted to 10. However, the linear correlation coefficient for the reaction without pH adjustment was 0.91, while that for the reaction with pH adjustment was only 0.84, implying that such a pH was not favorable for the synthesis of Ag-Au alloy NPs.

To verify these results, computer simulations were performed to assess whether the real UV–Vis spectra corresponded with theoretical calculations. The simulated UV–Vis spectra showed an extinction efficiency of the synthesized product. The relation of extinction, scattering, and absorption efficiency factors are as follows:

1 $$ {Q}_{\mathrm{ext}}={Q}_{\mathrm{sca}}+{Q}_{\mathrm{abs}} $$

The simulation methods of calculating the absorption and scattering efficiency usually belong to two categories: exact and approximated solutions [25]. For precise, spherically symmetric targets, such as homogenous spheres and multilayered concentric spheres, the Mie theory [26], the very first exact solution of Maxwell equations, can be applied. To date, several Mie theory computer codes have been developed [27]. Herein, the MieLab code [28], a free software specially designed for computing optical properties of multilayered spheres, was used. The theoretical basis of MieLab has been illustrated by Yang [29]. Users should provide initial parameters, including the number of layers, size distribution of each layer, complex refractive index tables for each layer, and refractive index of ambient medium, among others.

For homogeneous Ag-Au alloy spheres, the dielectric constants can be calculated as follows:

2 $$ {\varepsilon}_{\mathrm{Alloy}}\left({\chi}_{\mathrm{Ag}},\omega \right)={\chi}_{\mathrm{Ag}}{\varepsilon}_{\mathrm{Ag}}\left(\omega \right)+\left(1-{\chi}_{\mathrm{Ag}}\right){\varepsilon}_{\mathrm{Au}}\left(\omega \right), $$

where *χ*_Ag_ is the Ag fraction in the Ag-Au alloy, *ω* is the frequency of incident light, and *ε*_Ag_ and *ε*_Au_ are the dielectric constants for Ag and Au, respectively. The relationship between dielectric constant and refractive index is described in Eq. 3, where *n* is the complex refractive index.

3 $$ \varepsilon \left(\omega \right)=n{\left(\omega \right)}^2 $$

**Fig. 3:**
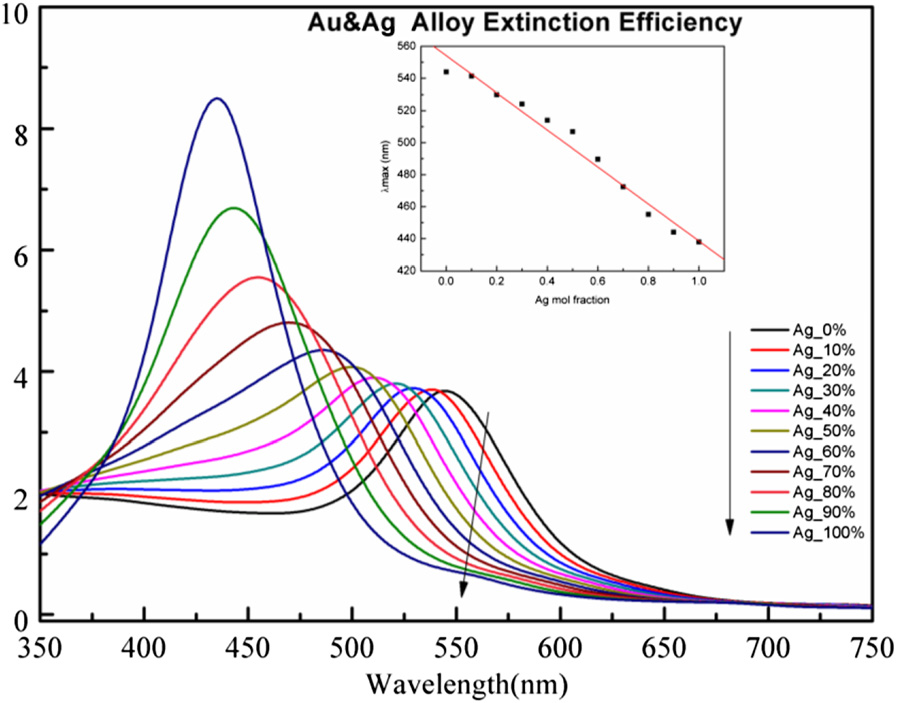
Simulated UV–Vis spectrum of Ag-Au alloy with different Ag contents by discrete dipole approximation. *Inset*: linear relationship between maximum absorption wavelength and Ag mole fraction

By simply calculating the dielectric constant of the Ag-Au alloy, we obtained its refractive index table as the input file. Initial parameters were set as number of layers = 1, radius = 10 nm, and refractive index of ambient medium = 1.4466. The simulation results are shown in Fig. 3. As the Ag fraction increased from 0 to 100 %, there was a significant increase in intensity, along with a blueshifting of the extinction maximum. Each curve shows only one peak, shifting from 537 (pure gold) to 415 nm (pure silver).

We compared UV–Vis absorption spectra of the prepared samples with simulated data for different Ag fractions of the Ag-Au alloy. The simulated spectrum indicated that Ag-Au alloy NPs only presented one plasmon resonance band and UV–Vis absorption wavelength shifts to shorter wavelengths with an increase in Ag fraction from 0 to 100 %; the linear correlation coefficient for the simulated spectrum was 0.97. Thus, experimental data was consistent with simulated data. Nevertheless, contrary to experimental data, the intensity of the UV–Vis absorption for the Ag fraction from 0 to 100 % in the simulated data gradually increased.

The existence of a single absorption band and a good linear relationship between the plasmon resonance bands with the increasing molar ratio of Ag confirmed the formation of Ag-Au alloy NPs. The mechanism of alloy formation with varying Ag:Au molar ratios was evaluated through analysis of the UV–Vis absorption spectra (Fig. 1). When HAuCl_4_ was added, the 405-nm plasmon resonance band quickly disappeared for the materials synthesized with Ag^+^:AuCl_4_^−^ ratios from 1:2 to 1:0.2, while a 387-nm new absorption peak appeared for the sample with a Ag^+^:AuCl_4_^−^ ratio of 1:0.125. The PSSMA-Ag NP solution was a yellowish brown color, indicative of the presence of Ag NPs, which rapidly changed to light yellow color following the addition of HAuCl_4_ in agreement with the results of previous studies [7, 8, 10, 11, 30]. This color change was attributed to an oxidation-reduction reaction of AuCl_4_^−^, given that the reduction potential of AuCl_4_^−^ to Au (1.498 V) is higher than that of Ag^+^ to Ag (0.799 V); this also led to the disappearance of the 405-nm peak. The stoichiometric equivalent of Ag(0) to AuCl_4_^−^ is 3 following a complete reduction reaction. Thus, when the Ag^+^:AuCl_4_^−^ ratio is above 1:3, a reduction from Au^3+^ to Au^2+^, Au^+^, and subsequently Au easily occurs. Nevertheless, the reduction of Au^3+^ to Au^2+^ and Au^+^ yields no absorptions in the UV–Vis region [16], thus explaining why the UV absorption peak of AgNPs disappeared without the appearance of any Au NP peaks upon addition of HAuCl_4_.

For the reaction at a Ag^+^:AuCl_4_^−^ ratio of 1:0.125, the solution turned from yellowish brown to a pale brown color following the addition of HAuCl_4_ to the PSSMA-Ag NPs solution. In addition, the emergence of an absorption peak at 387 nm could be attributed to a decrease in Ag NP size, since the Ag:AuCl_4_^−^ molar ratio was less than 1:3, and therefore Ag NPs could not be completely oxidized to Ag^+^ given the low AuCl_4_^−^ concentrations.

**Fig. 4:**
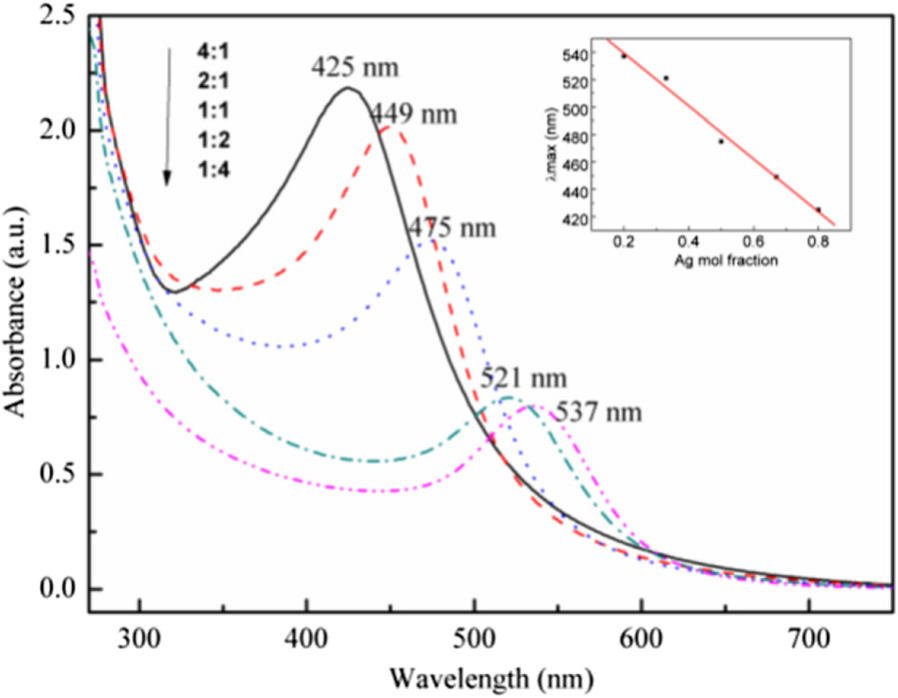
Concentration-related UV–Vis spectra of Ag-Au alloy heated at 95 °C in an autoclave for 19 h. The total concentration of PSSMA-Ag nanoparticles and HAuCl_4_ was 0.5 mM

A set of seed-mediated growth experiments was conducted at a fixed total concentration of PSSMA-Ag NPs and HAuCl_4_ of 0.5 mM (Fig. 4). A linear correlation coefficient of 0.99, comparing the maximum absorption wavelength to Ag content, was obtained. The absorption intensity of samples with Ag^+^:AuCl_4_^−^ ratios of 4:1, 2:1, 1:1, 1:2, and 1:4 gradually increased with an increasing Ag concentration. The intensity increase was consistent with the simulated Ag-Au alloy results, thus confirming the formation of an Ag-Au alloy.

### Optical Properties and DDA Simulation of Ag-Au Core-Shell NPs

**Fig. 5:**
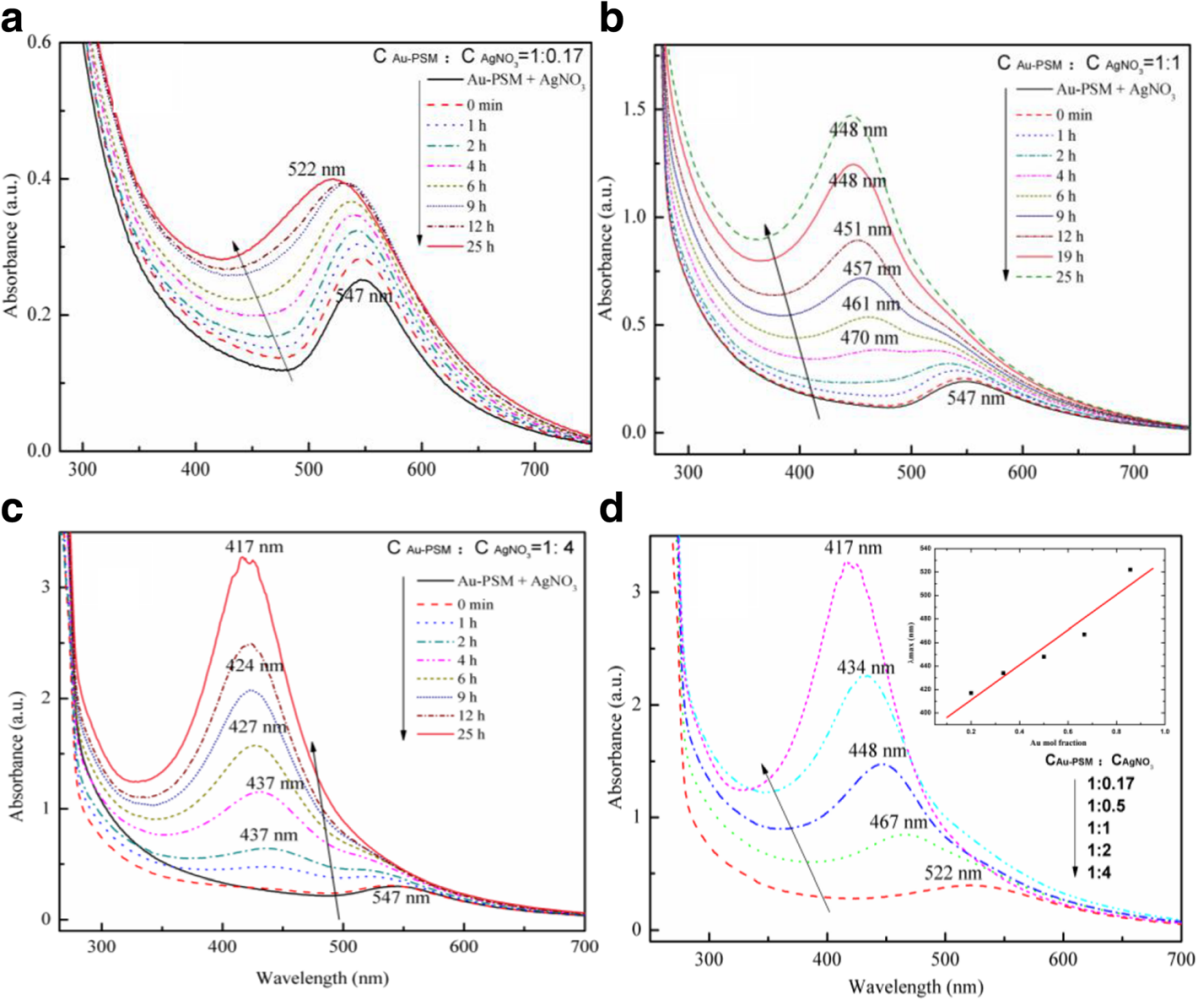
Time-dependent UV–Vis spectra of the prepared samples at different C_PSSMA-Au NPs_:C_AgNO3_ ratios **a** 1:0.17, **b** 1:1, and **c** 1:4 at a PSSMA-Au nanoparticle concentration of 0.25 mM. **d** Wavelength corresponding to the maximum absorbance for different C_PSSMA-Au NPs_:C_AgNO3_ ratios

PSSMA-stabilized Ag-Au core-shell NPs were prepared by heating the mixtures of PSSMA-Au NPs, and AgNO_3_ at 90 °Cas high temperature in synthesis of Ag-Au core-shell nanoparticles leads to nonuniform the sizes of NPs. UV–Vis absorption spectra for the prepared samples with Au/Ag molar ratios of 1:0.17, 1:0.5, 1:1, 1:2, and 1:4 are shown in Fig. 5. The Ag-Au bimetallic nanostructures with an Au:Ag molar ratio of 1:0.17 had only one absorption band in the 522–547-nm absorption range during the heating process. Samples with an Au:Ag molar ratio of 1:1 and 1:4 showed two plasmon resonance bands during the early stage of the reaction (Fig. 5b, c). After heating for several hours, only one peak was observed and attributed to the increasing thickness of the Ag shell.

After heating for the same amount of time, UV–Vis absorption bands of the five samples were located at 522, 467, 446, 434, and 417 nm and were blueshifted with the increasing Au:Ag ratio. The linear correlation coefficient for the maximum absorption wavelength versus Ag concentration was 0.91 and much lower than that of the Ag-Au alloy NPs (Fig. 5d). Thus, the possibility of alloy NP formation is excluded. On the other hand, the appearance of two plasmon resonance bands and the consistent blueshifted absorptions which eventually merged into one peak strongly prove the formation of core-shell Ag-Au NPs [6].

**Fig. 6:**
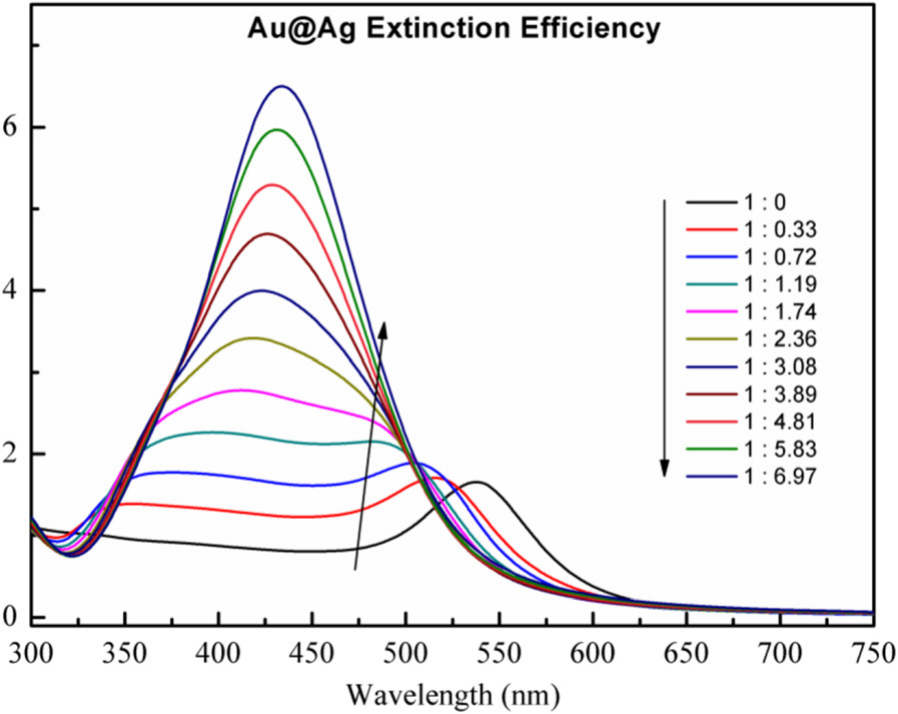
UV–Vis spectrum of Ag-Au core-shell nanoparticles simulated by the discrete dipole approximation model

To further analyze the synthesized core-shell NPs, UV–Vis absorption spectra of the prepared samples were compared with the simulated data (Fig. 6) for different Ag concentrations in Ag-Au core-shell NP formation.

For the simulation results of citric acid-coated Ag@Au spheres, all the particles were immersed in cyclohexane, leading to a three-layered sphere model. According to TEM images of the materials, the initial parameters were set at an Au core size of 532 nm. In the simulated model, as the Au:Ag^+^ ratio progressed from 1:0 to 1:2.36, the spectrum showed two characteristic peaks. With increasing particle size, the plasmon resonance bands gradually blueshifted and the absorption intensity increased with the increasing Ag ratio until only one peak could be observed, consistent with the experimental results obtained (Fig. 5). Changes in the peak positions and absorption intensity in both experimental and simulated spectra were influenced by an increase in Ag shell thickness around Au NPs following an increase in the Ag ratio. When the Au:Ag molar ratio was less than 1:0.33, only one absorption peak was observed. With a relative increase in Ag, two plasmon resonance bands appeared, representing the typical plasmon resonance absorption of Ag-Au core-shell NPs as described by Murphy et al. [6]. Finally, with high Ag concentrations, only the characteristic peak of Ag could be observed. The reducing ability of Au NPs is weaker than that of Ag NPs, and therefore Au NPs could not be oxidized by Ag^+^ to form Ag NPs when AgNO_3_ was added to the Au seed suspension. In fact, Ag NPs were formed by PSSMA reduction of AgNO_3_ on the surface of the Au seed through electrostatic adsorption.

### Morphological Study of Ag-Au Bimetallic Nanostructures

**Fig. 7:**
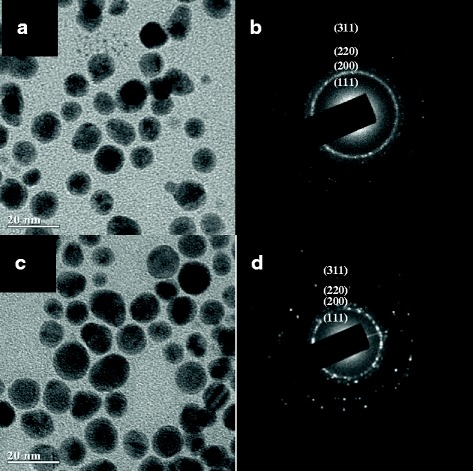
Transmission electron microscopy images and selected area electron diffraction patterns of the synthesized Ag-Au alloy nanostructures with C_PSSMA-Ag NPs_:C_HAuCl4_ of 1:0.5 (**a**, **b**) and 1:0.2 (**c**, **d**). The concentration of PSSMA-Ag nanoparticles was 0.14 mM

Representative TEM images of the PSSMA-stabilized metal NPs indicating a particle size of approximately 20 nm are shown in Fig. 7. The sample morphology varied according to the Ag content. The mechanism of formation of the alloy involves etching of the Ag NP surface by HAuCl_4_ before Au NP deposition on the Ag NP surface, followed by PSSMA reduction of Ag ions. The alloy process is incomplete and only prominent at the interface between the Ag core and Au shell [31]. Several lattice planes could be observed in their corresponding selected area electron diffraction patterns (Fig. 7b, d). Since Ag and Au have very similar lattice parameters and are miscible over the entire composition range [13, 32], the two series of lattice planes were practically identical.

Ag-Au alloy NPs were also synthesized in an autoclave with variation in the Ag:AuCl_4_^−^ ratios. The seed Ag NPs were not well-distributed in size and had varied morphologies ranging from spherical to triangular or polygonal. The Ag seed diameters were approximately 9.7 nm (Additional file 1: Figure S1a). Following addition of HAuCl_4_ (Ag:AuCl_4_^–^ ratios were 1:0.25 and 1:1), the obtained alloy NPs were mainly spherical with the existence of a few triangular NPs. The NP diameters were approximately 10.6 and 11.2 nm, respectively (Additional file 1: Figure S1b and S1c). This increase in size was attributed to the formation of Ag-Au alloy NPs. A further increase in HAuCl_4_ concentration lead to the formation of irregular-shaped NPs with a decrease in mean diameter to 10.6 nm, probably due to the formation of small Au NPs, which did not form an alloy with Ag seeds.

Ag-Au core-shell nanostructures were observed under TEM. Nearly spherical core-shell NPs of approximately 15 nm were observed to coexist with moderate amounts of NPs smaller than 2 nm (Additional file 1: Figure S2). It is hypothesized that the smaller particles were pure Ag due to the reduction of Ag^+^ by PSSMA. Reduction of Ag^+^ on the Au NPs resulted in the formation of core-shell nanostructures, while nucleation and growth of Ag without the substrate may have led to the formation of the smaller Ag NPs. In addition, the Ag shell thickened with an increasing Ag^+^ to Au^3+^ ratio, consistent with the UV–Vis spectra results.

### Characterization of Structure and Composition of Ag-Au Bimetallic Nanostructures

**Fig. 8:**
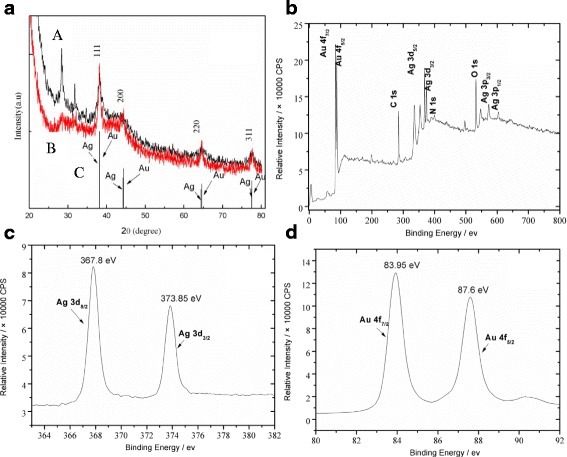
**a** Representative X-ray diffraction patterns of the PSSMA-Ag-Au alloy (*A* core-shell, *B* nanostructures, *C* standard stick patterns for fcc Ag or Au NPs). **b** XPS survey spectra of the Ag-Au alloy sample. **c**, **d** XPS high-resolution spectra of Ag 3d and Au 4f regions. The molar ratio of Ag:AuCl_4_^−^ was 1:2

X-ray diffraction patterns corresponding to the synthesized bimetallic NPs are shown in Fig. 8. Peaks at 38.2°, 44.4°, 64.5°, and 77.5° (Fig. 8a, b) can be indexed as the (111), (200), (220), and (311) lattice planes of the cubic Ag or Au nanostructures within experimental error [8, 32–34]. Since Ag and Au have very similar lattice parameters and complete miscibility for the composition range [13, 32, 33], these lattice planes are also observed in cubic Ag-Au alloy or core-shell structures. Further, the lattice plane of (111) is the most obvious, as it has a higher free energy and faster grow rate [35].

Additional evidence of the composition of Ag-Au alloy NPs was obtained by XPS. Figure 8b shows the XPS spectra of the Ag-Au alloy sample with an Ag:AuCl_4_^−^ molar ratio of 1:2. Peaks of C 1s, N 1s, and O 1s confirm the existence of a PSSMA-stabilized structure. Further, prominent peaks of Ag 3d and Au 4f regions, with a 6.0-eV difference between Ag 3d_5/2_ and 3d_3/2_, and 3.7-eV difference between Au 4f_7/2_ and 4f_5/2_, were recorded and attributed to the presence of Ag and Au atoms (Fig. 8c, d).

According to the XPS handbook, peaks at 368.3 and 374.3 eV corresponding to Ag 3d_5/2_ and Ag 3d_3/2_ were assigned to pure Ag and those at 367.5 and 373.5 eV to Ag_2_O. The binding energies of Ag 3d_5/2_ and Ag 3d_3/2_ of the alloy nanostructures with a Ag:AuCl_4_^−^ ratio of 1:2 observed herein were similar to those stated for Ag and Ag_2_O [34]. The 0.5-eV energy shift (Ag:AuCl_4_^−^ = 1:2) for the Ag peaks was attributed to the interaction between the carboxyl oxygen of PSSMA and the Ag core. For samples with Ag:AuCl_4_^−^ ratios of 1:0.5 and 1:0.33, a slight binding energy difference of 0.2 eV deviation from the standard values was observed, which suggests the formation of Ag-Au alloy NPs as reported in the literature [36].

## Conclusions

Water-soluble Ag-Au bimetallic NPs were synthesized by using polymer PSSMA as both a reducing agent and a stabilizer. By adjusting the Ag:Au molar ratio, different optical properties were observed during the formation of Ag-Au alloy and core-shell NPs. One plasmon resonance band of the Ag-Au alloy NPs could be adjusted within the range 422–517 nm by varying the HAuCl_4_ concentration in the aqueous PSSMA-stabilized Ag NP solutions. Due to the co-reduction of Ag and Au salts in PSSMA aqueous solution, UV–Vis absorption bands were observed to redshift with an increase in Au content. However, two plasmon resonance bands were observed during the formation of the Ag-Au core-shell NPs, with a blueshift of the UV–Vis absorption bands due to the reduction of Ag salts on the surface of Au seeds. Furthermore, changes in the Ag:Au ratio had an effect on the composition and morphology of the Ag-Au alloy and core-shell nanostructures.

## Additional File

Additional file 1: Figure S1. Transmission electron microscopy images and corresponding particle sizes. Histograms of the synthesized Ag NPs (a and b) and Ag-Au alloy nanostructures with C_PSSMA-Ag NPs_:C_HAuCl4_ of 1:0.25 (c and d) and 1:1 (e and f). The concentration of PSSMA-Ag nanoparticles was 0.14 mM. The size distribution of the particles was calculated from the TEM images of the prepared samples. **Figure S2.** Transmission electron microscopy images of different magnification factor of the as-prepared nanoparticles with C_PSSMA-Au NPs_:C_AgNO3_ of 1:0.5 (a and b) and 1:2 (c and d). The concentration of PSSMA-Au nanoparticles was 0.25 mM. (DOC 1549 kb)

## Abbreviations

DDA: discrete dipole approximation
NPs: nanoparticles
PSSMA: poly(4-styrenesulfonic acid-co-maleic acid)
PSSMA-Ag NPs: PSSMA-stabilized Ag nanoparticles
PSSMA-Au NPs: PSSMA-stabilized Au nanoparticles
TEM: transmission electron microscopy
XPS: X-ray photoelectron spectra

## References

[1] Hong S, Choi Y, Park S (2011). Shape control of Ag shell growth on Au nanodisks. Chem Mat.

[2] Wilson OM, Scott RWJ, Garcia-Martinez JC, Crooks RM (2005). Synthesis, characterization, and structure-selective extraction of 1-3-nm diameter AuAg dendrimer-encapsulated bimetallic nanoparticles. J Am Chem Soc.

[3] Wang C, Peng S, Chan R, Sun SH (2009). Synthesis of AuAg alloy nanoparticles from core/shell-structured Ag/Au. Small.

[4] Kumar GVP, Shruthi S, Vibha B, Reddy BAA, Kundu TK, Narayana C (2007). Hot spots in Ag core-Au shell nanoparticles potent for surface-enhanced Raman scattering studies of biomolecules. J Phys Chem C.

[5] Liu JH, Wang AQ, Chi YS, Lin HP, Mou CY (2005). Synergistic effect in an Au-Ag alloy nanocatalyst: CO oxidation. J Phys Chem B.

[6] Mallin MP, Murphy CJ (2002). Solution-phase synthesis of sub-10 nm Au-Ag alloy nanoparticles. Nano Lett.

[7] Yang J, Lee JY, Too HP (2005). Core-shell Ag-Au nanoparticles from replacement reaction in organic medium. J Phys Chem B.

[8] Shin Y, Bae IT, Arey BW, Exarhos GJ (2008). Facile stabilization of gold-silver alloy nanoparticles on cellulose nanocrystal. J Phys Chem C.

[9] Ah CS, Do Hong S, Jang DJ (2001). Preparation of AucoreAgshell nanorods and characterization of their surface plasmon resonances. J Phys Chem B.

[10] Jin YD, Dong SJ (2003). One-pot synthesis and characterization of novel silver-gold bimetallic nanostructures with hollow interiors and bearing nanospikes. J Phys Chem B.

[11] Zhang X, Tsuji M, Lim S, Miyamae N, Nishio M, Hikino S, Umezu M (2007). Synthesis and growth mechanism of pentagonal bipyramid-shaped gold-rich Au/Ag alloy nanoparticles. Langmuir.

[12] Gheorghe DE, Cui LL, Karmonik C, Brazdeikis A, Penaloza JM, Young JK, Drezek RA, Bikram M (2011). Gold-silver alloy nanoshells: a new candidate for nanotherapeutics and diagnostics. Nanoscale Res Lett.

[13] Link S, Wang ZL, El-Sayed MA (1999). Alloy formation of gold-silver nanoparticles and the dependence of the plasmon absorption on their composition. J Phys Chem B.

[14] Cheng LC, Huang JH, Chen HM, Lai TC, Yang KY, Liu RS, Hsiao M, Chen CH, Her LJ, Tsai DP (2012). Seedless, silver-induced synthesis of star-shaped gold/silver bimetallic nanoparticles as high efficiency photothermal therapy reagent. J Mater Chem.

[15] Hodak JH, Henglein A, Giersig M, Hartland GV (2000). Laser-induced inter-diffusion in AuAg core-shell nanoparticles. J Phys Chem B.

[16] Gonzalez CM, Liu Y, Scaiano JC (2009). Photochemical strategies for the facile synthesis of gold-silver alloy and core-shell bimetallic nanoparticles. J Phys Chem C.

[17] Hubenthal F, Ziegler T, Hendrich C, Alschinger M, Trager F (2005). Tuning the surface plasmon resonance by preparation of gold-core/silver-shell and alloy nanoparticles. Eur Phys J D.

[18] Chen JY, Wiley B, McLellan J, Xiong YJ, Li ZY, Xia YN (2005). Optical properties of Pd-Ag and Pt-Ag nanoboxes synthesized via galvanic replacement reactions. Nano Lett.

[19] Sun YG, Xia YN (2002). Shape-controlled synthesis of gold and silver nanoparticles. Science.

[20] Sinzig J, Quinten M (1994). Scattering and absorption by spherical multilayer particles. Appl Phys A-Mater Sci Process.

[21] Udayabhaskararao T, Sun Y, Goswami N, Pal SK, Balasubramanian K, Pradeep T (2012). Ag7Au6: a 13-atom alloy quantum cluster. Angew Chem Int Ed.

[22] Tong L, Cobley CM, Chen JY, Xia YN, Cheng JX (2010). Bright three-photon luminescence from gold/silver alloyed nanostructures for bioimaging with negligible photothermal toxicity. Angew Chem Int Ed.

[23] Song JM, Chen WT, Hsieh KH, Kao TH, Chen IG, Chiu SJ, Lee HY (2014). An in situ study on the coalescence of monolayer-protected Au-Ag nanoparticle deposits upon heating. Nanoscale Res Lett.

[24] Cai LJ, Wang M, Hu Y, Qian DJ, Chen M (2011). Synthesis and mechanistic study of stable water-soluble noble metal nanostructures. Nanotechnology.

[25] Zhao J, Pinchuk AO, McMahon JM, Li SZ, Ausman LK, Atkinson AL, Schatz GC (2008). Methods for describing the electromagnetic properties of silver and gold nanoparticles. Acc Chem Res.

[26] Mie G (1908). Beiträge zur Optik trüber Medien, speziell kolloidaler Metallösungen. Ann Phys.

[27] Du H (2004). Mie-scattering calculation. Appl Opt.

[28] Ovidio P, Pablo P, Umapada P (2011). MieLab: a software tool to perform calculations on the scattering of electromagnetic waves by multilayered spheres. Int J Spectrosc.

[29] Yang W (2003). Improved recursive algorithm for light scattering by a multilayered sphere. Appl Opt.

[30] Sun L, Luan WL, Shan YJ (2012). A composition and size controllable approach for Au-Ag alloy nanoparticles. Nanoscale Res Lett.

[31] Pedersen DB, Wang SL, Duncan EJS, Liang SH (2007). Adsorbate-induced diffusion of Ag and Au atoms out of the cores of Ag@Au, AIJ@Ag, and Ag@AgI core-shell nanoparticles. J Phys Chem C.

[32] Devarajan S, Bera P, Sampath S (2005). Bimetallic nanoparticles: a single step synthesis, stabilization, and characterization of Au-Ag, Au-Pd, and Au-Pt in sol-gel derived silicates. J Colloid Interface Sci.

[33] Pal A, Shah S, Devi S (2008). Preparation of silver-gold alloy nanoparticles at higher concentration using sodium dodecyl sulfate. Aust J Chem.

[34] Deng ZW, Chen M, Wu LM (2007). Novel method to fabricate SiO(2)/Ag composite spheres and their catalytic, surface-enhanced Raman scattering properties. J Phys Chem C.

[35] Zhang JT, Li XL, Sun XM, Li YD (2005). Surface enhanced Raman scattering effects of silver colloids with different shapes. J Phys Chem B.

[36] Han SW, Kim Y, Kim K (1998). Dodecanethiol-derivatized Au/Ag bimetallic nanoparticles: TEM, UV/VIS, XPS, and FTIR analysis. J Colloid Interface Sci.

