# Supplementary material for: Synthesis, Study, and Discrete Dipole Approximation Simulation of Ag-Au Bimetallic Nanostructures
Source: Nanoscale Res Lett. 2016 Apr 19;11:209. doi: 10.1186/s11671-016-1435-4 (PMC4837194; doi:10.1186/s11671-016-1435-4)
Supplement: Additional file 1: Figure S1. — Transmission electron microscopy images and corresponding particle sizes. Histograms of the synthesized Ag NPs (a and b) and Ag-Au alloy nanostructures with CPSSMA-Ag NPs:CHAuCl4 of 1:0.25 (c and d) and 1:1 (e and f). The concentration of PSSMA-Ag nanoparticles was 0.14 mM. The size distribution of the particles was calculated from the TEM images of the prepared samples. Figure S2. Transmission electron microscopy images of different magnification factor of the as-prepared nanoparticles with CPSSMA-Au NPs:CAgNO3 of 1:0.5 (a and b) and 1:2 (c and d). The concentration of PSSMA-Au nanoparticles was 0.25 mM. (DOC 1549 kb) [file 11671_2016_1435_MOESM1_ESM.doc]

**Electronic Supplementary Material**

Synthesis, study, and discrete dipole approximation

simulation of Ag-Au bimetallic nanostructures

Yang Hu^1^, An-Qi Zhang^2^, Hui-Jun Li^1^, Dong-Jin Qian^1^ and Meng Chen^1*^

^1^ Department of Chemistry, Shanghai Key Laboratory of Molecular Catalysis and Innovative Materials, Fudan University, Shanghai 200433, P. R. China.

^2^ Department of Materials Science, Fudan University, Shanghai 200433, P. R. China.

*Corresponding author: [chenmeng@fudan.edu.cn](mailto:chenmeng@fudan.edu.cn)


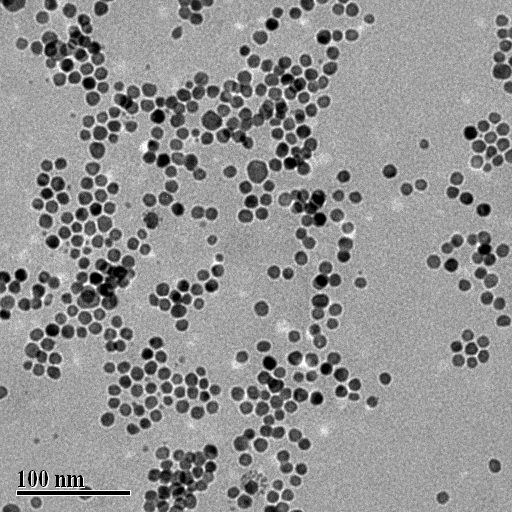


c


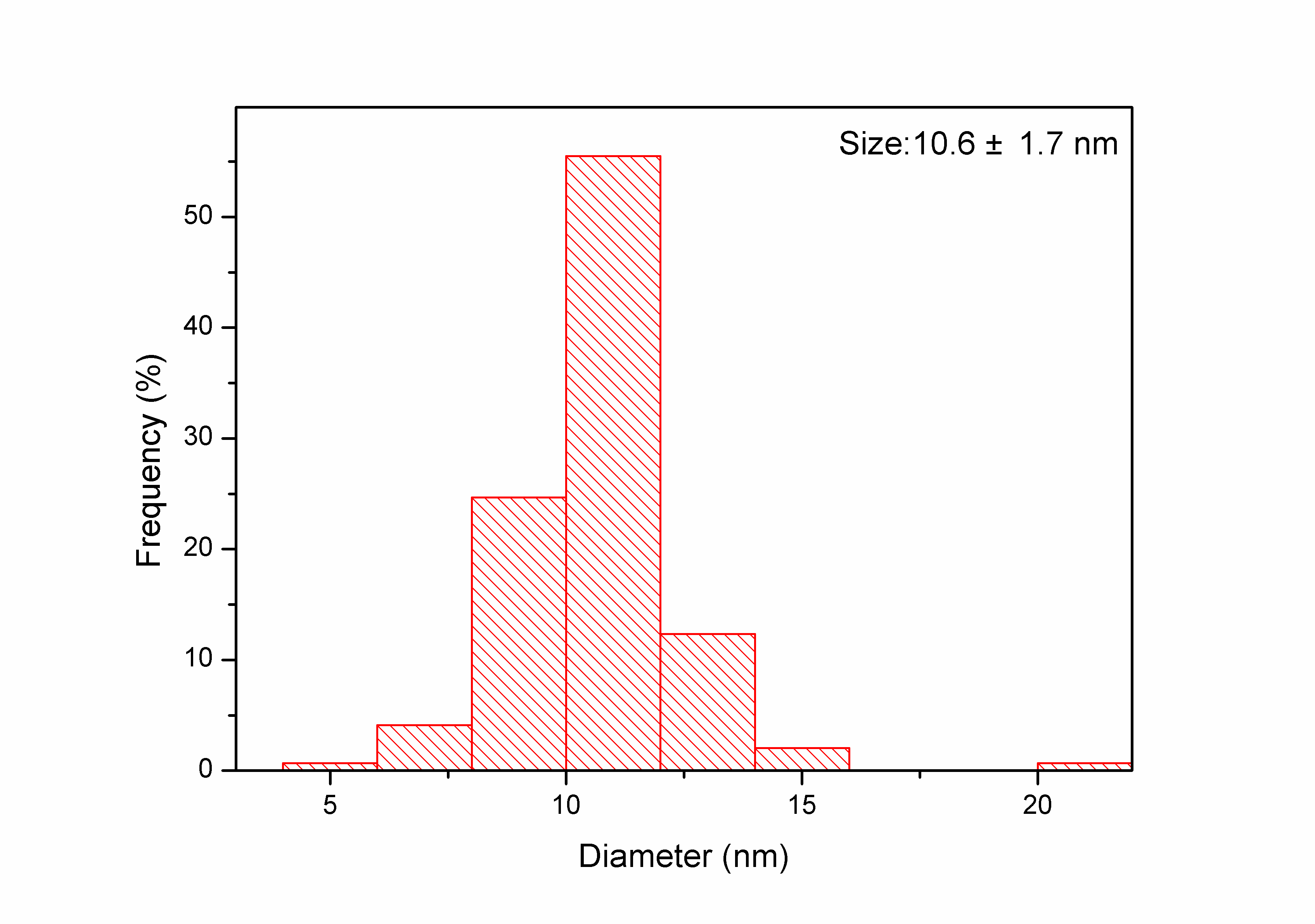


d


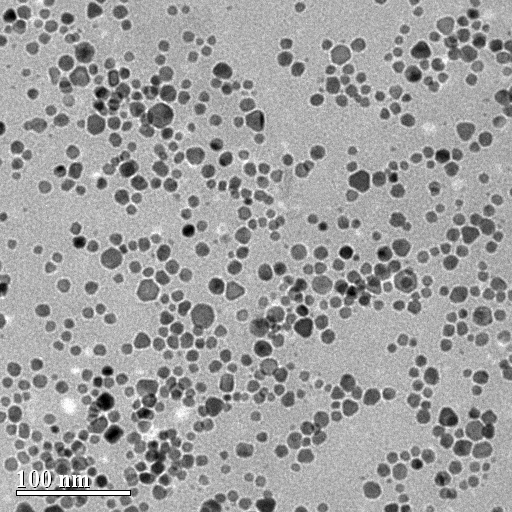


a


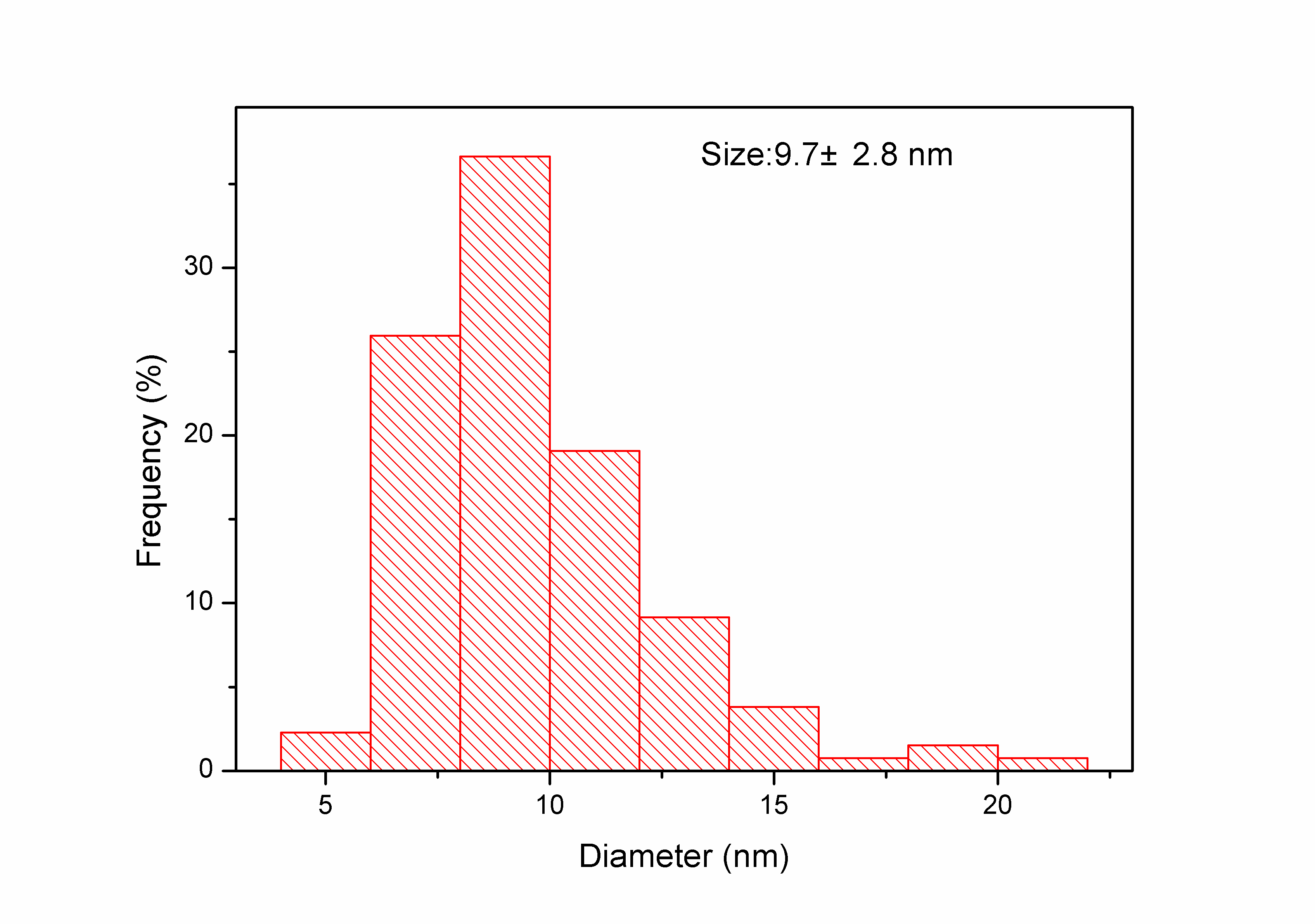


b


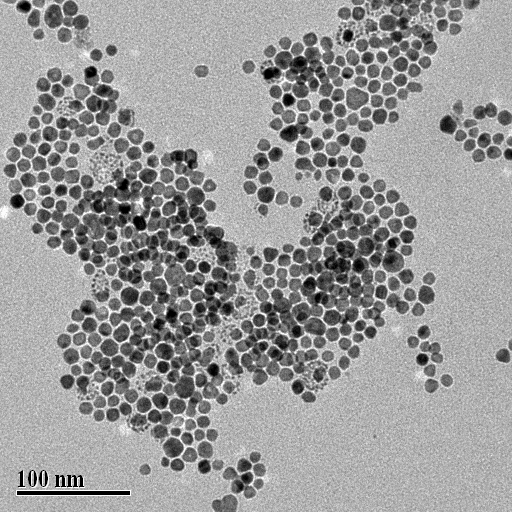


e


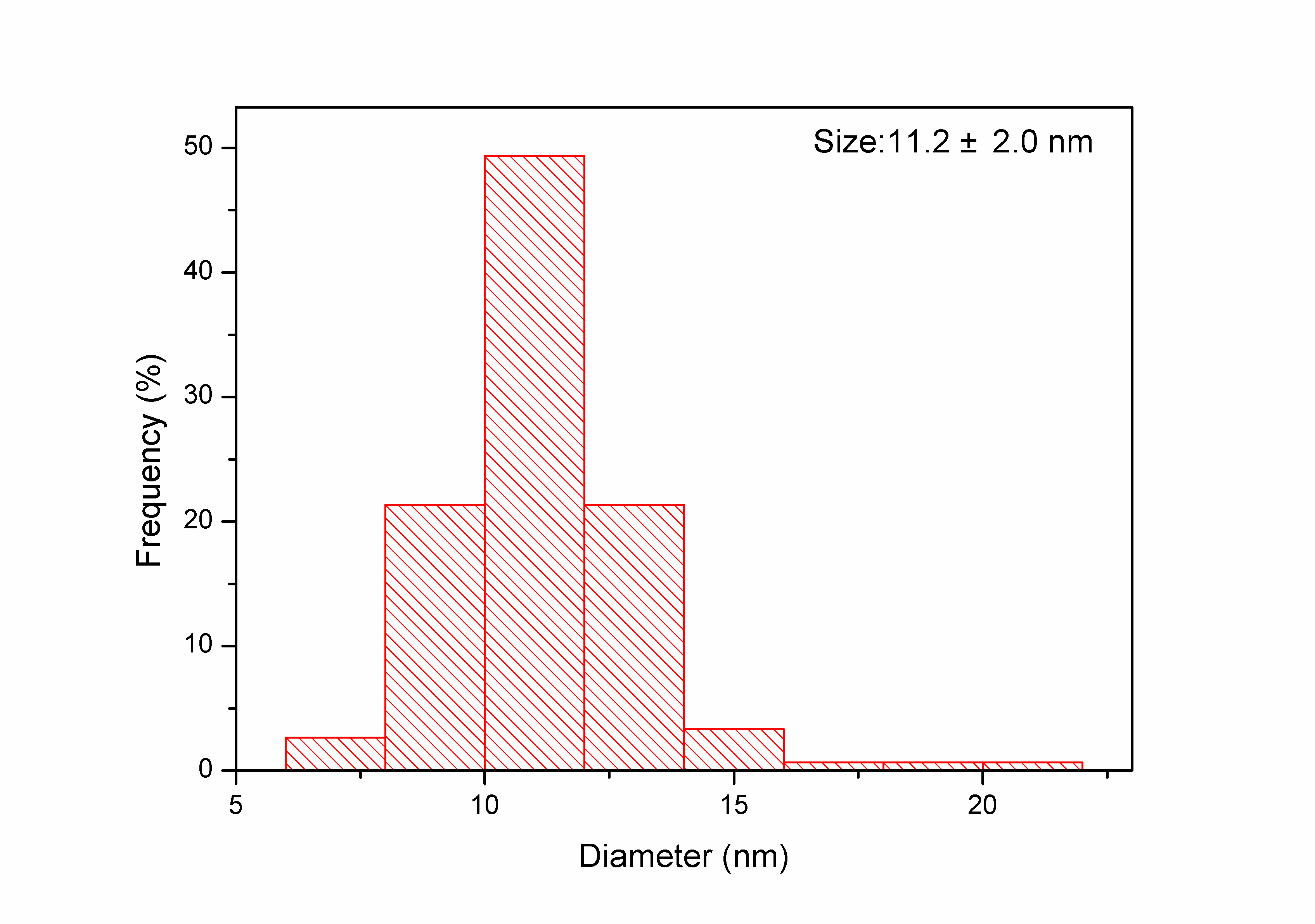


f

**Figure S1.** Transmission electron microscopy images and corresponding particle sizes Histograms of the synthesized Ag NPs (a and b) and Ag-Au alloy nanostructures with C_PSSMA-Ag NPs_:C_HAuCl4_ of 1:0.25 (c and d) and 1:1 (e and f).The concentration of PSSMA-Ag nanoparticles was 0.14mM. The size distribution of the particles was calculated from the TEM images of the prepared samples.


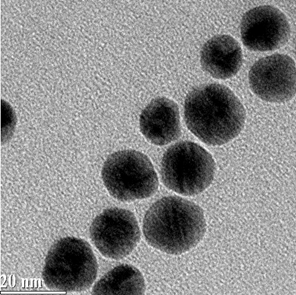


**d**


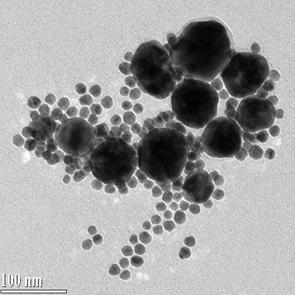


**c**


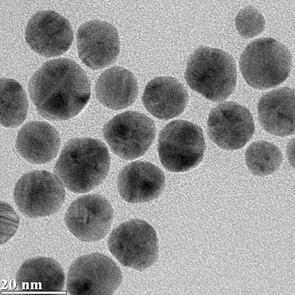


**b**


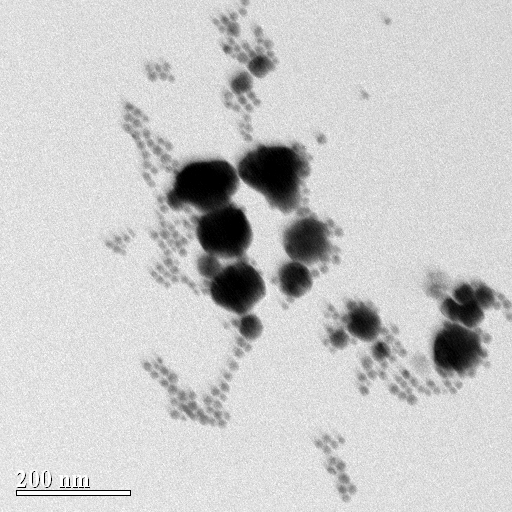


**a**

**Figure S2.** Transmission electron microscopy images of different magnification factor of the as–prepared nanoparticles with C_PSSMA-Au NPs_:C _AgNO3_ of 1:0.5 (a and b) and 1:2 (c and d). The concentration of PSSMA-Au nanoparticles was 0.25 mM.
